# Paris terrorist attack: early lessons from the intensivists

**DOI:** 10.1186/s13054-016-1246-0

**Published:** 2016-04-08

**Authors:** TRAUMABASE Group

**Affiliations:** Hôpital Beaujon, Département d’Anesthésie Réanimation, 100 boulevard du Général Leclerc, 92118 Clichy, Cedex France

## Abstract

During the night of 13–14 November, the city of Paris was exposed, within a few hours, to three bomb explosions, four shooting scenes, and one 3-hour hostage-taking of several hundred people causing at least 130 deaths and more than 250 injured victims. Most unstable patients were transferred to the six trauma centers of the Paris area, all members of the TRAUMABASE Group. A rapid adaptation of the organization of trauma patients’ admittance was required in all centers to face the particular needs of the situation. Everything went relatively well in all centers, with overall hospital mortality below 2 %. Nevertheless, most physicians nowadays agree that anticipation, teaching, and training are crucial to appropriately face such events. All of us have learned many additional issues from this experience. Following a meeting of the TRAUMABASE Group, the most relevant issues are detailed in the following.

## Organization

The initial team was quite easily identified from human resources already present within the hospital. They had to be quickly split into two groups: those finishing the ongoing routine procedures, and those organizing the forthcoming tasks. A single director of medical operations (DMO), with recognized authority, was quickly identified in each department. Their first mission was to recall medical resources, taking simultaneous care of the anticipated evolution and the unavoidable needs of teams for the day after. All centers have noticed an important feeling of frustration in people asked to stay at home to rest. This must be considered in parallel with the massive mobilization of everyone to help in one way or another. The recall of nonmedical resources was performed in consultation with the crisis teams of hospital administrations as anticipated by the “*Plan blanc*” which is dedicated to this purpose. The attack occurring at night was fortunate. It is easier to mobilize unused resources than to manage the rapid ending of numerous surgical procedures, which would have been required during the day.

Most of us encountered difficulties with patients’ identities. Severe patients were anonymous, without any identity documentation left at the shooting scene. Effective systems exist outside and within the hospital but do not communicate. The required time to check the coherence between different naming systems was not available. This highlighted the importance of being simultaneously able to integrate an unambiguous identity established outside and able to generate a temporary identity devoid of ambiguity. A person dedicated to this mission is probably required.

The DMOs following tasks were mainly to dedicate appropriate teams for each patient and then to distribute patients among resources according to both information provided by the teams and monitoring of the global context. Consultation with surgical teams was of varying quality depending on the center. In the military hospital, a couple of senior intensivists/surgeons planned for immediate in-hospital triage. In several civilian hospitals, regulatory principles of the massive influx of patients had to be explained at this point among doctors, stressing the importance of good preparation. All centers have planned or achieved a multidisciplinary debriefing meeting. Out-of-hospital information tracking was crucial and has been difficult for all DMOs. Resources including spare operating rooms were necessary in case of an expected new wave of patients but many patients already present had to be operated on relatively urgently. This highlights the importance of a communication channel between the police authorities, out-of-hospital emergency services, and DMOs. In the absence of software devoted to this function, placard papers or erasable boards were essential tools. They allowed monitoring by everyone of the status and position of each patient. They avoided having to distinguish patients mostly by their lesions, with nearly all presenting almost identical lesions. Stabilized patients were redirected to the intensive care unit or to another area, offloading the reception area.

## Medical aspects

Damage control, which is routinely used in trauma patients, aims to treat within <45 minutes any life-threatening lesions delaying the treatment of secondary, nonvital, wounds [1]. In case of resource weakness, damage control offers an additional benefit by quickly redistributing resources to other patients. In other words, when a great wave of patient admittance is anticipated, stabilized patients having to be treated must wait mainly to avoid resource limitation. At 1:00 a.m. on 14 November, 30 minutes following the onset of the police assault of the Bataclan concert hall, the treatment of nonvital surgical lesions had to be delayed but the overall time finally available for surgery allowed avoiding restrictions in indications or limitations of the quality of surgical procedures. Medical procedures and computed tomography (CT) scans were limited everywhere to those which were absolutely and immediately necessary. Most CT scans were done after the event. Catheterizing was delayed until the operating room. When needed, vasoconstrictors were administered on peripheral routes. CT scans were performed only in patients whose support depended on them.

## Conclusion

As expected, the TRAUMABASE Group’s conclusion is that daily trauma training associated with specific massive patient influx teaching would further enhance the support that we have experienced as satisfactory.

## Abbreviations

CT: Computed tomography
DMO: Director of medical operations
